# Are Birth-preparedness Programmes Effective? Results From a Field Trial in Siraha District, Nepal

**Published:** 2006-12

**Authors:** Robert A. McPherson, Neena Khadka, Judith M. Moore, Meena Sharma

**Affiliations:** ^1^ Save the Children-USA, Himalayan Field Office, GPO Box 2218, Kathmandu, Nepal

**Keywords:** Pregnancy, Delivery, Obstetric care, Parturition, Safe motherhood, Behaviour, Behaviour change communication, Communication, Community, Community health services, Interpersonal relations, Counselling, Delivery of healthcare, Health services, Nepal

## Abstract

The birth-preparedness package (BPP) promotes active preparation and decision-making for births, including pregnancy/postpartum periods, by pregnant women and their families. This paper describes a district-wide field trial of the BPP implemented through the government health system in Siraha, Nepal, during 2003–2004. The aim of the field trial was to determine the effectiveness of the BPP to positively influence planning for births, household-level behaviours that affect the health of pregnant and postpartum women and their newborns, and their use of selected health services for maternal and newborn care. Community health workers promoted desired behaviours through inter-personal counselling with individuals and groups. Content of messages included maternal and newborn-danger signs and encouraged the use of healthcare services and preparation for emergencies. Thirty-cluster baseline and endline household surveys of mothers of infants aged less than one year were used for estimating the change in key outcome indicators. Fifty-four percent of respondents (n=162) were directly exposed to BPP materials while pregnant. A composite index of seven indicators that measure knowledge of respondents, use of health services, and preparation for emergencies increased from 33% at baseline to 54% at endline (p=0.001). Five key newborn practices increased by 19 to 29 percentage points from baseline to endline (p values ranged from 0.000 to 0.06). Certain key maternal health indicators, such as skilled birth attendance and use of emergency obstetric care, did not change. The BPP can positively influence knowledge and intermediate health outcomes, such as household practices and use of some health services. The BPP can be implemented by government health services with minimal outside assistance but should be comprehensively integrated into the safe motherhood programme rather than implemented as a separate intervention.

## INTRODUCTION

The Ministry of Health and Population (MoHP) in Nepal has confronted high rates of maternal and infant mortality during the past two decades through various approaches. The safe motherhood plan of the MoHP defines a range of complementary interventions to improve maternal and newborn health, one of which is the birth-preparedness package (BPP). The purpose of the package is to encourage pregnant women, their families, and communities to plan for normal pregnancies, deliveries, and postnatal periods and to prepare to deal effectively with emergencies if they occur. The BPP is a demand-creation intervention that promotes key messages and behaviour change via inter-personal communication through community health volunteers.

Birth-preparedness programmes generally address ‘three delays’ to care-seeking for obstetric emergencies—delay in recognition of problem, delay in seeking care, and delay in receiving care at facility. These delays represent barriers that often result in preventable maternal deaths (1–4). The presence of a skilled birth attendant (SBA) at delivery is recognized as essential to preventing maternal mortality (5, 6). A strategy to reduce the three delays should begin at the community level and be linked to improving access to basic/comprehensive essential obstetric care (BEOC/CEOC) (1, 2, 6, 7).

Birth-preparedness, an established concept, is recommended consistently as a best practice; however, few studies have examined the effectiveness of a comprehensive BPP and its associated costs (4, 8). While there is no universal definition of birth-preparedness, many packages that address birth-preparedness promote the following: (a) preparation for normal birth by selecting a SBA and place of delivery; (b) preparation of essential items for delivery, such as a clean delivery-kit; (c) knowledge of danger signs for mother and newborn and when to seek help; (d) knowledge of where and to whom to go for help; (e) arranging access to funds and means for emergency transportation and medical care; and, (f) prior identification of blood donors (4).

A review of available reports yielded several examples of projects that have promoted birth-preparedness, although few reported the results of rigorous evaluations. The MotherCare Project included birth-planning interventions and focused on planning for emergencies (4). The CHANGE Project developed a maternal survival toolkit that included standard elements of birth-preparedness but also addressed neonatal care, the need for early postnatal care, and issues surrounding HIV/AIDS (4, 7). JHPIEGO developed tools for improving communication and collaboration among stakeholders to advocate for essential maternal care (9). The Home Based Life Saving Skills package focuses on the family and community level to increase access to lifesaving care and to decrease delays in reaching referral facilities. The package has been tested in India and Ethiopia with promising results in increasing preventive behaviours and birth-preparedness among women fully exposed to the project; however, there was little evidence in the India trial that care-seeking following emergencies had been improved following the project (10, 11). A birth-preparedness intervention in Dinajpur, Bangladesh, substantially increased the use-rate of emergency obstetric services; 45% of families in the project area reported that they had access to community-support systems (12, 13). The Prevention of Maternal Mortality Programme (1987–1997) found that inadequate funds and transport were key causes of delay in deciding to seek care and in reaching facilities (4).

The Saving Newborn Lives (SNL) Initiative viewed collaboration with the MoHP on the BPP as an opportunity to integrate the promotion of essential newborn-care behaviours into the safe motherhood programme. The SNL Initiative, therefore, supported the conduct of a district-wide field trial of the BPP in Siraha district in Nepal from 2002 to 2004. This paper presents the results of the study. The birth-preparedness/complication readiness (BP/CR) matrix contributed to the study design. This matrix, which was previously used in Nepal as part of the SUMATA (care, share, and prepare) communication initiative, delineates the roles of policy-makers, facility managers, providers, communities, and families in improving maternal and newborn care and outlines plans and actions that can be implemented by each group (9).

## MATERIALS AND METHODS

### Description of programme

Siraha, a predominantly rural district in the eastern *terai* (plains) of Nepal, is administratively divided into 106 rural Village Development Committees (VDCs) and two municipalities—Lahan and Siraha. Twelve health posts and 89 sub-health posts provide primary healthcare services to rural communities, while two public hospitals and three primary healthcare centres provide higher-level services to the general population. The majority of the population of 615,000 belong to ethnic groups indigenous to the *terai*, live in extended families, and engage in subsistence farming. Although estimates of infant mortality rate (IMR) in Siraha are unavailable, the 2001 Demographic and Health Survey reports IMR estimates of 81 in the *terai* and 78 in the Eastern Development Region of Nepal (14). The maternal mortality rate in Nepal is estimated at 539 (15). Siraha has not received inputs from any major donor-supported programmes that specifically target safe motherhood. The target populations of the BPP programme (herein after Programme) were 145,000 women, aged 15–49 years, who live in Siraha and their 24,000 newborns. The goal of the Programme was to improve the health and survival of mothers and newborns by increasing knowledge of community members and practice of beneficial household behaviours and by increasing the use of maternal and newborn health services.

The BPP consists of a framework of content and procedures that are elaborated in the guidelines that the community health workers (CHWs) use for structuring inter-personal communications with clients, a flip-chart to be used by the CHWs, and key chains that are given to each pregnant woman. Messages in the flip-chart and key chain focus on four areas of birth-planning: antenatal care; care for the mother and newborn during and after delivery; danger signs in women and newborns; and financial and logistical preparations for pregnancy, delivery, and the postnatal period. These topic areas, taken together, represent the Programme's definition of birth-preparedness. The key chain—a symbol of woman's empowerment in Nepal—consists of laminated cards that repeat the messages and illustrations contained on the flip-chart. The flip-chart and key chain were pre-tested for understanding with both literate and illiterate women.

The implementation of the Programme commenced in March 2003. The CHWs who implemented the BPP included Mobilizers (Female Community Health Volunteers [FCHVs] and trained traditional birth attendants (TBAs)) and Supporters (Maternal Child Health Workers [MCHWs] and Village Health Workers [VHWs]). The Mobilizers and Supporters were each trained for two days on counselling techniques for small groups and individuals and the use of BPP tools. The Supporters received three additional days of training on technical aspects of safe motherhood. The Supporters were supposed to visit each Mobilizer every month and assess their work using a check-list; in practice, the Mobilizers were supervised once every 3–4 months. The FCHVs organized monthly discussions in mothers’ groups attended by women of all ages and used flip-charts to communicate the BPP messages. The FCHVs, members of mothers’ groups, and trained TBAs also identified pregnant women and contacted them and their family members—including husbands and, to a lesser extent, fathers-in-law—through individual counselling sessions. Facility-based CHWs counselled women who use facility-based services. All family members were encouraged to be present at delivery and accompany pregnant woman/mother in the case of emergency.

The Siraha District Health Office (DHO) was involved at all levels of the implementation of the Programme. The BPP-related tasks were part of the workplan of the DHO, monitoring-data were collected through the reporting system of the DHO, supervision of the BPP was integrated into the supervisory activities of DHO, and the DHO staff served as Master Trainers for the roll-out of the BPP.

The Programme was implemented with minimal external inputs aside from modest financial and technical support from the SNL Initiative for activities, such as training, community meetings, materials, and evaluation.

### Statistical methods

#### Observational subjects

Quantitative data were collected through the baseline and endline surveys conducted in September 2002 and September 2004 respectively. Survey respondents were defined as mothers of live infants aged less than one year at the time of survey. The activities of the Programme commenced in March 2003. The endline-survey respondents potentially were exposed to the Programme throughout a minimum of the final six months of their pregnancies. [The endline survey included respondents who gave birth in (at the earliest) September 2003. The last six months of pregnancies of women who delivered in September 2003, therefore, fell during the implementation period of the Programme in Siraha.]

A multi-stage 30-cluster design was used for selecting 300 respondents for each survey. Each VDC in Siraha district is divided into nine wards. In each survey, 30 wards were selected using the probability-proportional-to-estimated-size (PPES) methodology from a sample frame of all wards (in 95 of 106 VDCs) and both the municipalities in Siraha. The remaining nine VDCs were excluded due to security considerations. The sample frames at baseline and endline were identical, although the samples of wards were independent. The Valley Research Group conducted the baseline survey using its own surveyors. The SNL Initiative staff and consultants oversaw the conduct of the endline survey and used community-level government health staff due to security considerations; the surveyors did not survey communities where they were posted.

The survey teams entering a cluster began by identifying geographically-definable communities (i.e. segments) within the ward along with an approximation of the number of households within each segment. One segment was then selected using the PPES methodology. A list of all households in the selected segment was then prepared. Following the random selection of a starting household from the list of households, the surveyors visited the starting household to determine if an eligible respondent lived there. The surveyors then proceeded to the next closest house, and the next closest, etc., identifying and interviewing eligible respondents, until 10 eligible respondents in the cluster were successfully interviewed.

#### Statistical techniques

Tests of statistical significance and calculation of odds ratios and confidence intervals were performed using the logistic and svymean statistical routines respectively on Stata version 7.0. The logistic routine was used for determining if the difference between the baseline and the endline variable estimate was statistically significant when adjusted for independent variables that include age of mother, age of infant, literacy status of mother, employment status of mother, and birth-registration status of infant. Adjustments were made for the cluster design using the psu(variable) option for all routines.

### Ethical considerations

The Programme involved only promotional health messages; it was, therefore, unnecessary to obtain ethical clearance from the National Health Research Council in Nepal for the conduct of the Programme and household surveys. Participation of observational subjects in the Programme activities and in the baseline and endline surveys was voluntary. All procedures followed in the study were in accordance with the Helsinki Declaration.

## RESULTS

The field trial of the BPP measured the process and intermediate outcome variables that included exposure to the BPP messages, change in knowledge and practices, use of services, and reaction to emergencies. The results are detailed below.

### Characteristics of the sample

The characteristics of the sample from the baseline and endline surveys are presented in Table 1. The differences between the two samples among the variables in the table were not statistically significant except for the variable caste/ethnicity of mother.

**Table 1.** Characteristics of the household survey sample

Variable	Baseline	Endline	p value
Age (months) of infant	5.9	5.4	0.15
Percentage of infants whose birth was registered	22.1	16.0	0.16
Age (years) of mother	24.4	24.5	0.77
Percentage of mothers who work outside home	46.2	50.3	0.48
Percentage of mothers who are literate	25.8	19.7	0.15
Caste/ethnicity of mother (%t)			
**Brahmin**	2	0	
Chhetri	0	1	
Chaudhari (Tharu)	10	4	
Damai/Kami/Sarki	34	27	0.00*
**Gurung/Magar/Lama**	0	0	
Muslim	4	10	
Yadav	20	19	
Mushahar	15	18	
Other	15	21	

*Pearson chi-square (8)=33.01

### Exposure to BPP messages

**Fig. 1.**
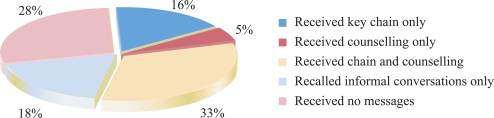
Direct and indirect exposures of mothers to BPP messages, Siraha district: endline survey estimates, September 2004

Two measures of exposure to the BPP messages were defined: (a) formal exposure, i.e. a respondent who received a key chain and/or was counselled using a BPP flip-chart; and (b) exposure to specific BPP messages, i.e. a respondent who reported having heard a specific message promoted through the BPP. The results of the endline survey revealed that, after one and a half years of the implementation of the BPP, 49% of the respondents received a key chain, and 54% were formally exposed to the BPP. An additional 18% of the respondents reported no formal exposure but did report indirect exposure to the BPP messages through conversations with family members or other community members (Fig. 1).

BPP=Birth-preparedness package

Results of logistic regression analysis revealed that the variables (a) mother works outside the home (odds ratio [OR]=0.6; p=0.04) and (b) age of the newborn (one month increase in age: OR=1.1; p=0.01) were associated with formal exposure. Home-based mothers presumably had more time to interact with the FCHVs and mothers’ groups, where formal exposure takes place. It is not clear why mothers of older infants were more likely to be formally exposed to the BPP. Mothers of older infants might have more time to be exposed to the BPP as some exposure might have occurred post-delivery. Alternatively, this variable might be a proxy for the variable intervention period, suggesting that more mothers were formally exposed to the BPP in early 2004 than in mid-to-late 2004—perhaps due to shortages of key chains or other supplies during the latter stages of the implementation of the Programme. The following variables were included in the model and were non-significant: birth registered, age of mother, and literacy of mother.

The respondents of the endline survey were asked if they had heard the following three key BPP messages: (a) “A pregnant women should make four antenatal care visits with a trained health worker”; (b) “A newborn should be breastfed for the first time immediately after birth”; and, (c) “A mother and newborn should have their health checked by a trained health worker within days after birth”. The respondents who answered ‘yes’ were asked how they had been exposed. Exposure to the three messages ranged from 45% to 76%. Of respondents reporting exposure, 30–48% were exposed through the key chain, while 58–64% were exposed through a health worker.

### Qualitative information

Peer key-informant monitoring was used for gauging the acceptability and understanding of the BPP messages. Key-informant mothers-in-law and pregnant women reported that most ‘women like us’ understood the messages and found them to be acceptable. These informants confirmed the survey results by reporting that key chains—FCHVs, trained TBAs, and other health workers—were important channels for receiving the BPP messages. They noted that some pregnant women prefer trained TBAs to FCHVs as Mobilizers because they also deliver services.

### Challenges to coverage

The Programme intended to supply a key chain to every pregnant woman. The number of key chains provided to the DHO did not account for women who were already pregnant at the start of the Programme, contributing to an insufficient supply of key chains.

The modes of inter-personal communication used for promoting the BPP messages also limited the coverage. The mothers’ groups represent the principal mechanism through which the FCHVs counsel mothers; however, many pregnant women do not participate in mothers’ groups for cultural and logistical reasons. Although the FCHVs were encouraged to promote the BPP messages through home-visits, these visits are not part of the job description of the FCHV, and some FCHVs were reluctant to perform this task. The coverage may have been influenced by the armed conflict between the government security forces and the insurgents.

### Changes in essential newborn care

**Fig. 2.**
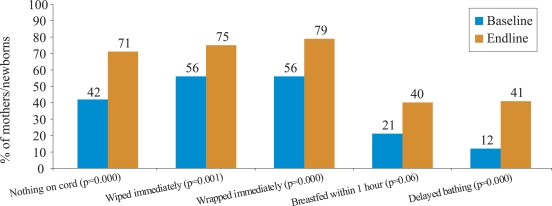
Changes in essential newborn care practices in BPP programme, Siraha district: baseline and endline survey estimates (September 2002 and September 2004)

The endline estimates for essential newborn practices promoted through the BPP increased by 20–30% compared to the baseline (Fig. 2). The use of a clean home delivery-kit or new/boiled blade to cut the umbilical cord was high at baseline (96%) and, thus, not intensely promoted during the Programme. [Contents of the kit included: razor blade, cord ties, soap, plastic sheet, plastic disc (to cut cord on), and instruction sheet.]

BPP=Birth-preparedness package

### The birth-preparedness index

The SUMATA project originally conceptualized and developed the birth-preparedness index (BPI). The Programme adopted the SUMATA definition of BPI to facilitate the comparison of the results of different BPP-related efforts in Nepal. The BPI is composed of seven discrete, equally-weighted variables that measure different aspects of the birth-preparedness process. The BPI is calculated at the level of the individual as the percentage of the following components that the mother reports regarding her most recent pregnancy/delivery: (a) received antenatal care at least once from a trained provider; (b) names prolonged labour as a danger sign during delivery; (c) names excessive bleeding as a danger sign during delivery; (d) made financial preparations for emergencies during pregnancy; (e) made preparations for emergency transportation during pregnancy; (f) delivery attended by a SBA; and, (g) received postpartum care from a trained provider within six weeks of delivery.

**Fig. 3.**
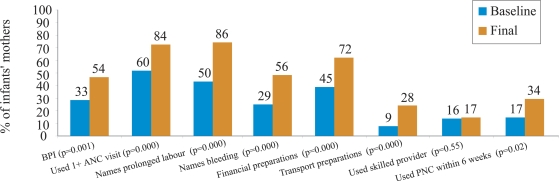
Changes in birth-preparedness index in BPP programme, Siraha district: baseline and endline surveys estimates (September 2002 and September 2004)

The BPI increased from 33% at baseline to 54% at endline. Increases in six of the seven components of the BPI were statistically and practically significant (Fig. 3). The use of a SBA did not increase.

ANC=Antenatal care;
BPI=Birth-preparedness index;
BPP=Birth-preparedness package;
PNC=Postnatal cate

### Skilled birth attendance

The use of a SBA at endline remained unchanged from baseline at 17%. Of 89% (266/300) of the endline survey respondents who stated that, using a SBA was ‘important’ or ‘very important’, only 18% (48/266) reported that a SBA attended their delivery. The 218 respondents who stated that the use of a SBA was important but did not use one were asked, “why did not a trained health worker attend your delivery?” Fifty-nine percent (129/218) of the respondents cited “do not think it was necessary” as the reason. Cost was the second most commonly-cited reason (16%; 36/218). Limited knowledge of who provides local skilled birth attendance services and a preference for home-deliveries likely contributed to the low use of SBAs, as “no service available nearby” and “no practice in the community” were frequently cited.

Key-informants in Siraha reported that many community members believe that SBAs are only necessary if an emergency occurs during delivery and, thus, only contact them in the event of a crisis. This study did not specifically explore the rationale underlying community preference for home-deliveries; however, it does not appear to be rooted in negative attitudes towards the use of health facilities. Both mothers-in-law and pregnant women reported that fathers-in-law were influential in taking decisions for issues relating to finance and transport—both related to the use of SBAs.

### Use of antenatal and postnatal care

A comparison of the baseline and endline estimates of antenatal and postnatal care services with a trained provider showed an increase for both the services. Attendance at two or more antenatal care visits increased from 49% to 73% (p=0.001). Attendance at four or more antenatal care visits was not measured at baseline and was 31% at endline. The use of postnatal care services within one week of delivery increased from 11% to 25% (p=0.01), while the use within six weeks of delivery doubled from 17% to 34% (p=0.02).

### Maternal tetanus toxoid, postpartum vitamin A and iron usage

The FCHVs in Siraha do not distribute vitamin A and iron to pregnant/postpartum women, while the coverage of baseline tetanus toxoid (TT) was moderately high, thus limiting the potential of the Programme to increase their use. The survey results showing no appreciable baseline-to-endline change in mothers’ use of postpartum iron and vitamin A were likely biased by the questionnaire design. The unbiased survey-based estimates of TT coverage showed no change between baseline (54%) and endline (57%). The health post staff reported intermittent shortages of commodities, a factor that may have contributed to these results.

### Care-seeking during emergencies

Of women who reported emergencies, the percentage who received treatment at a health facility remained constant at baseline and endline. For example, of women who reported emergencies during pregnancy, the percentage who received treatment changed little from 83% at baseline to 85% at endline (p=0.32). The corresponding indicator for care-seeking following emergencies during delivery and the postpartum period increased slightly from 59% to 62% (p=0.95) and from 78% to 85% (p=0.56) respectively.

### Association among exposure to messages, knowledge, and behaviour

The positive associations between the exposure to the BPP materials/messages and the knowledge or practice of the content of the BPP message can strengthen conclusions regarding the impact of the Programme. Logistic regression analysis was used for exploring the degree of association at endline between the exposure to the BPP (independent variable) and the BPP-related knowledge or service use/behaviour (outcome variables) for two messages: (a) made 4+ antenatal care visits and (b) practised immediate breastfeeding. Two measures of exposure—formal exposure and exposure to specific BPP messages—were employed. The analysis controlled for the demographic variables and the cluster sample design.

The results presented in Table 2 suggest a strong link between (a) the exposure to the messages and the correct knowledge and (b) the exposure to the messages and the correct behaviour/use of service. The level of influence of different sources of exposure appeared to be mixed. While both the definitions of exposure are positively associated with the variable “made 4+ antenatal care visits”, only one definition of exposure—exposure to message on immediate breastfeeding—was positively associated with practised immediate breastfeeding.

**Table 2.** Levels of association among exposure to messages, knowledge, and practice/use at endline

Behaviour/service	Baseline estimate	Endline estimate	Outcome variable	Independent variable	Associated variables* (odds ratio; p value)
			Knowledge	Formal BPP exposure	Mother is literate (2.4; 0.003)
			Knowledge	Exposure to messages	Exposed to message (4.9; 0.000)
					Mother is literate (2.2; 0.01)
Immediate breastfeeding after birth	21	40	Reported practice	Formal BPP exposure	Mother works outside home (1.9; 0.01)
					Age of mother=1 year greater (0.95; 0.03)
					Birth is registered (2.1; 0.03)
			Reported practice	Exposure to messages	Exposed to message (4.2; 0.000)
					Age of mother=1 year greater (0.96; 0.05)
					Birth is registered (2.3; 0.02)
			Knowledge	Formal BPP exposure	Formal exposure to BPP (4.4; 0.000)
			Knowledge	Exposure to messages	Exposed to message (29; 0.000)
					Age of mother=1 year greater (1.07; 0.04)
Made 4+ ANC visits	Not measured	31	Made 4+ ANC visits	Formal BPP exposure	Formal exposure to BPP (2.3; 0.007)
					Age of newborn=1 month greater (1.1; 0.02)
					Mother is literate (1.8; 0.04)
			Made 4+ ANC visits	Exposure to messages	Exposed to message (2.8; 0.02)
					Age of newborn=1 month greater (1.1; 0.004)

*The following independent variables were included in the regression model: exposure (either formal BPP exposure or exposure to message; age (months) of newborn; birth registered (yes/no); age (years) of mother; mother works outside home (yes/no); mother is literate (yes/no);

ANC=Antenatal care;

BPP=Birth-preparedness package

## DISCUSSION

The results of the BPP field trial in Siraha, Nepal, suggest that, while birth-preparedness programmes can serve as an important aspect of community mobilization and demand-side of the safe motherhood strategy, there are significant barriers that constrain individuals—particularly young women—who wish to practise behaviours and care-seeking outside conventional practice. Birth-preparedness programmes do hold promise to positively influence household-level behaviours and birth-planning and increase the use of selected maternal health services. The improvements in essential newborn practices are especially noteworthy and represent an important finding for Nepal where most births occur at home, and behaviours surrounding newborns were thought to be resistant to change. Furthermore, birth-preparedness activities can be implemented and sustained by government health services with minimal assistance from non-governmental organizations.

The study found that the level of association between different sources of exposure (i.e. formal exposure and exposure to specific BPP messages) and mothers’ correct knowledge and/or reported correct behaviours was mixed. This finding suggests that the influence of exposure to the BPP materials on knowledge and practices of mothers may differ depending on the message and that other sources of information may be as or more important than the BPP materials. Overall, these findings suggest that the Programme caused a positive change in selected outcome variables.

The positive results reported in this paper were achieved despite sub-optimal coverage, as only 54% of the mothers were directly exposed to the BPP materials. Rates of higher coverage may lead to further increases in the adoption of desired behaviours.

The contents of the BPP included both ‘old’ messages (e.g. use antenatal care) and ‘new’ topics (e.g. save for emergencies, identify blood donors). The BPP personnel reported that the BPP was helpful in arranging existing messages into a coherent framework. They noted that some new messages were difficult to promote because they lacked concrete, pragmatic mechanisms to achieve their performance. The broad focus of birth-preparedness messages should be matched by an equally broad array of activities to support their practice.

### Skilled birth attendance in Siraha

The low use of SBAs in Siraha results from factors that include the following: faith in a traditional system of delivery care that views delivery as a natural event that takes place at home; a view of modern health services and SBAs as a ‘last resort’ to be used only if an emergency develops; a cadre of poorly-trained SBAs who do not possess the requisite skills and who are generally unwilling and/or unable to attend births at home; a populace with inadequate information regarding who the SBAs in their areas are and how to access them; and high costs associated with emergency transport and health services. These conclusions are supported by other studies in South Asia (16, 17).

Demand-side programmatic efforts that provide households with information regarding the risks of childbirth need to recognize the gap that exists between knowledge of danger signs and problem recognition—thus contributing to the ‘first delay’—among both household members and TBAs (18). The community-based programme that seeks to shorten the first delay must go beyond merely increasing household members’ knowledge of danger signs. Programmes also need to focus on changing household perceptions of the susceptibility of mothers and newborns during delivery and the postpartum period—thus shortening the ‘second delay’. In India, Das Gupta found that a woman's relationship with and status within her family unit, her mobility, her educational level, and the place of birth (mother's house or mother-in-law's house) were critically associated with her ability to seek healthcare services (19). Birth-preparedness programmes must empower and enable mothers to communicate more effectively with other household members.

Options to arrange for a SBA to attend a birth in Siraha are limited. The MoHP defines community-based cadres, such as Auxiliary Nurse Midwife (ANM) and MCHW as SBAs, although they do not meet internationally-accepted criteria. An MCHW or ANM is posted in each of Siraha's 106 VDCs and is theoretically accessible within one-hour travel. In practice, the ANMs and MCHWs are seldom asked to attend births. Services provided by these workers are supposed to be free, although informal charges of US$ 10–20 may be levied. There is one government hospital in Siraha municipality that offers 24-hour delivery services but does not provide basic emergency obstetric services. Women in Siraha who require a caesarean section or management of severe complications have a choice of using one of two facilities offering CEOC services in Lahan, Siraha—the government hospital or a private nursing home—or travelling to a neighbouring district or India. Most women in Siraha must travel 2–8 hours by oxcart and/or taxi to access these services at a transportation cost alone of US$ 5–30. Borghi *et al.* noted that the average cost of a caesarean section in a representative cross-section of districts in Nepal—including service, transport, and opportunity costs, and additional charges—exceeds US$ 150. Thus, while the SBAs—as categorized by the Government—are relatively accessible, facilities and personnel that offer basic lifesaving services to a woman with an obstetric emergency are much less so.

The BPP messages must be consistent with available health services. The lack of supply-side programming in the Siraha BPP field trial represents a central limitation of the Programme design. Care-seeking decisions take place within a complex setting that includes community-level factors, such as characteristics of local health systems. Birth-preparedness programmes will be more effective if they improve accessibility to and quality of health services.

Our results suggest that a programme that merely encourages pregnant women to use SBAs will not achieve dramatically positive results in the Nepali context. Further research on the root causes of the low use of SBAs and innovative programming to overcome barriers are required to achieve progress. This research must go beyond the household to explore the health-system issues and determine what is required to establish reliable, sustainable delivery services that are acceptable to the community. Such research may yield unexpected results. The MIRA study in Makwanpur, Nepal, identified a preference among the general public for community-level treatment for newborns, suggesting that merely strengthening health facilities is unlikely to have a major impact on perinatal care-seeking practices (20).

### Alternative models of demand creation

Diversity is required in demand-creation strategies. The fundamental strategy of the BPP was inter-personal communications based on a traditional teaching/counselling relationship between the CHWs and the household members. Two other models of message-delivery have yielded promising results in Nepal. The MIRA trial achieved positive reductions in maternal and neonatal mortality through a participatory, action-learning communication strategy implemented by non-governmental personnel (21). The SNL Initiative supported the implementation of the BCC Project in Kailali, Nepal, that achieved gains in the practice of essential newborn practices equivalent to those achieved by the BPP (22). Under the BCC Project, the FCHVs promoted a focused set of messages through inter-personal communications that were supported by a multi-media campaign. Taken together with the results of the BPP field trial, these results suggest that inter-personal communications delivered through CHWs can be an effective core strategy to achieve a positive change in household-level behaviours within the safe motherhood field in Nepal. Creative testing of variations of this strategy is called for to maximize its cost-effectiveness.

## ACKNOWLEDGEMENTS

The Bill and Melinda Gates Foundation funded this work through the Saving Newborn Lives (SNL) Initiative grant awarded to Save the Children-USA. It was implemented jointly by the SNL Nepal staff and the District Health Office and staff of Siraha district supported by Family Health Division of the Ministry of Health and Population.

The SNL Initiative in Nepal has been implemented by Save the Children-USA (SC-USA) under a grant from the Bill and Melinda Gates Foundation. The SNL Initiative acknowledges the MoHP for the opportunity to conduct a field trial of the first-ever district-wide implementation of the BPP. The SNL Initiative acknowledges The Center for Development and Population Activities and other partners who collaborated in the BPP design. The SNL Initiative also acknowledges the Siraha DHO, without whose leadership and guidance the BPP programme could not have been successfully implemented.

The authors acknowledge the technical contributions provided by Dr. Eric Starbuck and Dr. Mary Taylor. Finally, the SNL Initiative expresses thanks to every BPP Mobilizer and Supporter.
